# Murine Typhus in Canary Islands, Spain, 1999–2015

**DOI:** 10.3201/eid2702.191695

**Published:** 2021-02

**Authors:** José María Robaina-Bordón, Cristina Carranza-Rodríguez, Michele Hernández-Cabrera, Margarita Bolaños-Rivero, Elena Pisos-Álamo, Nieves Jaén-Sánchez, Araceli Hernández-Betancor, Laura Suárez-Hormiga, José Luis Pérez-Arellano

**Affiliations:** Universidad de Las Palmas de Gran Canaria, Las Palmas de Gran Canaria, Spain (J.M. Robaina-Bordón, C. Carranza-Rodríguez, M. Hernández-Cabrera, E. Pisos-Álamo, N. Jaén-Sánchez, L. Suárez-Hormiga, J.L. Pérez-Arellano);; Hospital Universitario de Gran Canaria Doctor Negrín, Las Palmas de Gran Canaria (J.M. Robaina-Bordón);; Complejo Hospitalario Universitario Insular-Materno Infantil de Gran Canaria, Las Palmas de Gran Canaria (C. Carranza-Rodríguez, M. Hernández-Cabrera, M. Bolaños-Rivero, E. Pisos-Álamo, N. Jaén-Sánchez, A. Hernández-Betancor, L. Suárez-Hormiga, J.L. Pérez-Arellano)

**Keywords:** murine typhus, Rickettsia typhi, rickettsia, bacteria, endemic fleaborne typhus, infections, fever, neglected diseases, reemerging communicable diseases, zoonoses, Canary Islands, Spain

## Abstract

To document the epidemiology, clinical features, and outcomes of murine typhus patients in the Canary Islands (Spain), we analyzed data that were retrospectively collected for 16 years for 221 patients. Murine typhus in the Canary Islands is characterized by a high rate of complications (31.6%), mainly liver, lung, kidney or central nervous system involvement.

Murine typhus is a febrile disease caused by *Rickettsia typhi* (*1*). Rickettsia are obligate, intracellular, gram-negative bacilli that are transmitted to mammals by various arthropod vectors, including ticks, lice, mites, and fleas (*2*). The classic *R. typhi* life cycle involves rats of the subgenus *Rattus* (such as *R. rattus* and *R. norvegicus*) and their fleas (especially *Xenopsylla cheopis*). Adaptation to new reservoirs (cats, dogs, opossums) and vectors, in particular *Ctenocephalides felis* (cat flea), has probably led to the reappearance of murine typhus in industrialized countries (*3*).

Murine typhus remains a neglected disease despite its worldwide distribution. It is one of the most frequent causes of fever of intermediate duration (FID), defined as fever of 7–28 days, and is not associated with localizing signs or diagnostic clues after a complete evaluation in southern Spain and the Canary Islands (*4*,*5*). Underdiagnosis represents a major health cost because unnecessary diagnostic tests might be performed and treatment might be inadequate (*6*). Although it is considered a mild disease, a large number of patients require hospital admission and show development of life-threatening complications (*7*). Our aim was to document the epidemiology, clinical features, and outcome of murine typhus in the Canary Islands (Spain).

## ­The Study

The study included 221 adults >14 years of age who were inpatients and outpatients at the Hospital Universitario Insular of Las Palmas (Las Palmas de Gran Canaria, Spain), who received a diagnosis of murine typhus during June 1, 1999–December 31, 2015. Epidemiologic, clinical, and laboratory data were retrospectively collected from medical records. Diagnosis of murine typhus was based on detection of antibodies against *R. typhi* by using an indirect immunofluorescence test and 2 criteria. Criterion 1 was titer >1:1,280 for IgM in 1 sample, and criterion 2 was a 4-fold increase in IgG titers between 2 consecutive samples. A total of 72 (32.6%) patients were given a diagnosis according to criterion 1, and 149 (67.4%) patients were given a diagnosis according to criterion 2. Clinical and laboratory data for both groups were analyzed separately.

**Figure 1:**
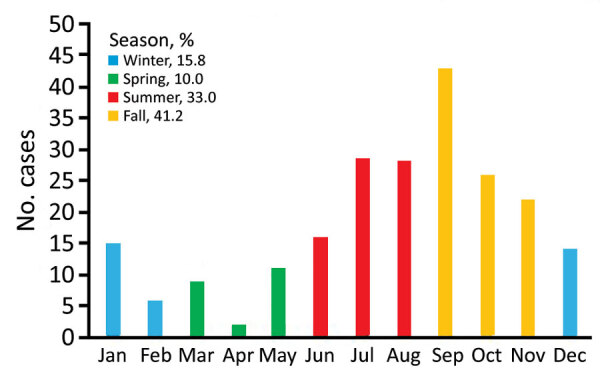
Monthly distribution of cases of endemic murine typhus, Canary Islands, Spain, 1999–2015.

**Figure 2:**
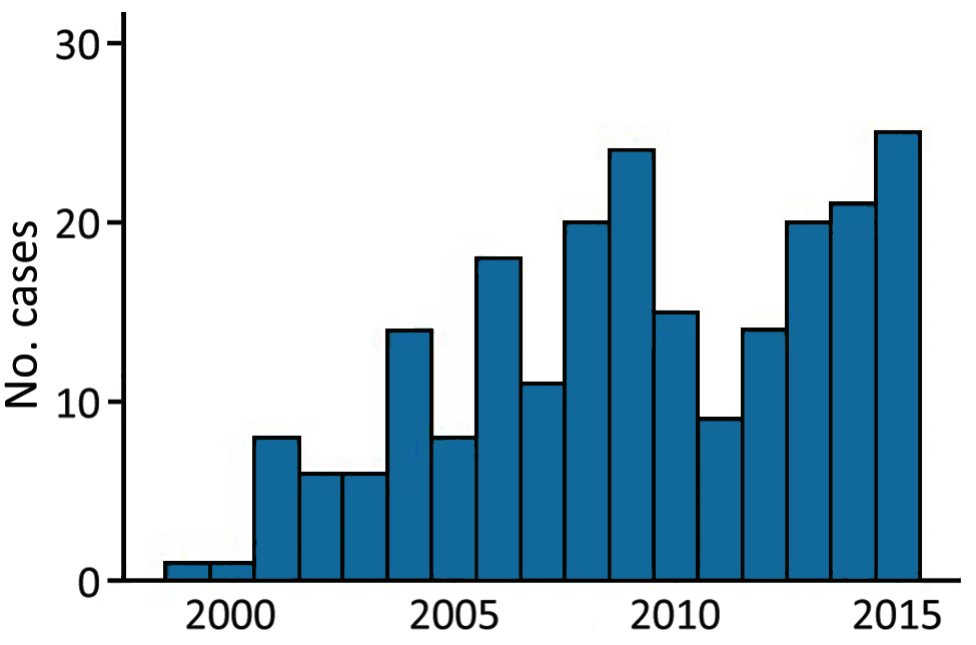
Annual distribution of cases of murine typhus, Canary Islands, Spain, 1999–2015.

Murine typhus was more frequent during July–November (Figure 1). The mean ± SD number of cases diagnosed per year was 18 ± 5.33. We provide the annual distribution of cases (Figure 2). Most (91.4%, 202/221) case-patients lived in urban areas; 73.3% (162/221) were male; and the median age was 40 years (interquartile range 28.5–52.5 years). Most (88.7%, 188/212) reported close contact with animals, especially dogs (66%, 140/212) and cattle (42%, 89/212). Arthropod bites were reported by 34 (19.5%) of 174 case-patients.

We provide the main clinical features recorded (Table 1). A total of 180 (82.95%) of 217 patients had high fever (median temperature 39.8°C) of >1 week duration. Pharyngitis was more frequently observed for younger (<20 years of age) patients (7/20, 35%) than for older patients (12/169, 7.1%) (p = 0.001). Rash was present more often in younger patients (8/19, 42.1%) than in older patients (42/190, 22.1%) (p = 0.05). We also provide laboratory findings (Table 2). Most (184/195, 94.4%) had urinalysis alterations in the form of microhematuria, proteinuria, or leukocyturia.

**Table 1:** Clinical findings for patients with cases of endemic murine typhus, Canary Islands, Spain, 1999–2015

Finding	Total, no. (%)	IgM >1:1,280, no. (%)*	4-fold IgG titer increase, no. (%)†	p-value
Symptom or sign				
Headache, n = 206	181 (87.9)	59 (88.1)	122 (87.8)	0.95
Sweating, n = 175	129 (73.7)	44 (74.6)	85 (73.3)	0.85
Myalgias, n = 186	135 (72.6)	39 (65)	96 (76.2)	0.11
Nausea/vomiting, n = 206	103 (50)	32 (47.8)	71 (51.1)	0.66
Dry cough, n = 200	79 (39.5)	23 (36.5)	56 (40.9)	0.56
Rash, n = 211	56 (26.5)	14 (21.2)	36 (25.2)	0.53
Abdominal pain, n = 200	45 (22.5)	15 (22.7)	30 (22.4)	0.96
Classic triad, n = 201‡	46 (22.9)	14 (21.5)	32 (23.5)	0.75
Conjunctivitis, n = 201	41 (20.4)	12 (18.8)	29 (21.2)	0.69
Diarrhea, n = 221	41 (18.6)	11 (15.3)	30 (20.1)	0.38
Odynophagia, n = 182	24 (13.2)	16 (13.1)	8 (13.3)	0.97
Tachycardia, n = 164	75 (45.7)	28 (54.9)	47 (41.6)	0.11
Hepatomegaly, n = 209	37 (17.7)	12 (17.9)	25 (17.6)	0.96
Relative bradycardia, n = 162§	21 (13)	3 (5.9)	18 (16.2)	0.07
Pharyngitis, n = 189	19 (10.1)	8 (12.7)	11 (8.7)	0.39
Lymphadenopathy, n = 198	16 (8.1)	10 (15.6)	6 (4.5)	0.01
Splenomegaly, n = 209	17 (8.1)	5 (7.5)	12 (8.5)	0.81
Altered pulmonary auscultation, n = 210	17 (8.1)	7 (10.3)	10 (7)	0.42
Flea bite, n = 93	6 (6.5)	0 (0)	6 (9.4)	0.17
Costovertebral angle tenderness, n = 199	11 (5.5)	6 (9.2)	5 (3.7)	0.18
Jaundice, n = 210	11 (5.2)	4 (5.9)	7 (4.9)	0.75

*Patients given a diagnosis by testing of 1 sample (criterion 1).  †Patients given a diagnosis by testing of 2 consecutive samples (criterion 2).  ‡Fever, headache and rash.  §Temperature >38.9°C and heart rate <110 beats/min in the absence of treatment with β-blockers.

**Table 2:** Laboratory test results for patients with cases of endemic murine typhus, Canary Islands, Spain, 1999–2015*

Finding	No. (%)	IgM >1:1,280, no. (%)†	4-fold IgG titer increase, no. (%)‡	p-value	Cutoff values
Anemia, n = 220	38 (17.3)	16 (22.5)	22 (14.8)	0.15	<12 g/dL Hb in women, <13 g/dL Hb in men
Leukopenia, n = 220	23 (10.5)	7 (9.9)	16 (10.7)	0.26	<4,000/μL
Leukocytosis, n = 220	42 (19.1)	18 (25.4)	24 (16.1)	0.26	>11,000/μL
Thrombocytopenia, n = 218	127 (58.3)	28 (39.4)	99 (67.3)	<0.01	<150,000/μL
Increased ESR, n = 106	64 (60.4)	27 (65.9)	37 (56.9)	0.42	Upper limit of normality calculated according to age and sex
Prolonged PT, n = 197	82 (41.6)	27 (42.9)	55 (41)	0.81	<80%
Increased aPTTr, n = 188	6 (3.2)	2 (3.4)	4 (3.1)	0.99	>1.2
Plasma Cr increase, n = 215	47 (21.9)	16 (23.5)	31 (21.1)	0.72	>1.2 mg/dL
Hyponatremia, n = 205	119 (58)	30 (50)	89 (61.4)	0.13	<135 mEq/L
Plasma CK increase, n = 97	20 (20.6)	5 (13.5)	15 (25)	0.17	>232 U/L
Plasma urea increase, n = 211	36 (17.1)	12 (18.5)	24 (16.4)	0.72	>40 mg/dL
Plasma LDH increase, n = 138	131 (94.9)	40 (90.9)	91 (96.8)	0.21	>190 U/L
Plasma ALT increase, n = 197	184 (93.4)	59 (92.2)	125 (94)	0.76	>35 U/L
Plasma AST increase, n = 196	182 (92.9)	55 (88.7)	127 (94.81)	0.14	>35 U/L
Plasma AP increase, n = 109	38 (34.9)	10 (25.6)	28 (40)	0.15	>136 U/L
Plasma GGT increase, n = 166	91 (54.8)	29 (52.7)	62 (55.9)	0.74	>85 U/L
Microhematuria, n = 195	146 (74.9)	45 (72.6)	101 (75.9)	0.6	>5 RBCs/mm^3^
Proteinuria, n = 195	169 (86.7)	54 (87.1)	115 (86.5)	0.9	Positive urine test strip
Leukocyturia, n = 194	123 (63.4)	38 (62.3)	85 (63.9)	0.83	>10 WBCs/mm^3^

*ALT, alanine aminotransferase; AP, alkaline phosphatase; aPTTr, activated partial thromboplastin time ratio; AST, aspartate aminotransferase; Cr, creatinine; CK, creatine kinase; ESR, erythrocyte sedimentation rate; GGT, γ-glutamyl transpeptidase; Hb, hemoglobin; LDH, lactate dehydrogenase; PT, prothrombin Time; RBCs, red blood cells; WBCs, white blood cells. †Patients given a diagnosis by testing of 1 sample (criterion 1).  ‡Patients given a diagnosis by testing of 2 consecutive samples (criterion 2).

Complications developed in 31.6% (68/215) of patients, especially hepatitis (22/221, 10.0%), acute renal failure (21/215, 9.8%), meningitis (12/215, 5.6%), and pneumonia (9/215, 4.2%). No differences were found between patients given a diagnosis by using criterion 1 or 2 (Appendix). Cerebrospinal fluid samples from patients who had meningitis were characterized by a clear appearance, moderate mononuclear pleocytosis (range 6–43 cells), mild proteinorachia, and standard glucose levels. Round pneumonia developed in 2 patients and retinitis in 1 patient. 

A total of 51 (22.6%) of the 221 patients were hospitalized: 29 (56.9%) had complications, 12 (23.5%) experienced vomiting, and 10 (19.6%) needed a diagnostic workup. The average length of hospital stay was short (median 6 days; interquartile range 4–9 days). Seven patients did not receive treatment because of spontaneous recovery. The remaining patients received doxycycline. Two patients required admission to the intensive care unit because of multiple organ failure, and no patients died. Low transient IgM titers and no IgG titers against other microorganisms were found in admission serum samples, especially against *Coxiella burnetii* (36/218, 16.5%) and Epstein-Barr virus (13/218, 6.0%). 

## Conclusions

Murine typhus was diagnosed primarily in middle-age men and showed a similar male:female ratio as in other clinical series (*8**)*. The seasonal prevalence of murine typhus during late summer and fall has been described (*7*). This temporal pattern seems to be related to the increased propagation activity of the vector linked to higher temperatures. The number of annual cases is similar to that reported by others (*9**–**11*), and diagnoses increased over the study period. However, these data probably underestimate the incidence of murine typhus because of the absence of clinical hallmarks and the fact that this disease is self-limiting.

The clinical features of murine typhus observed in this study are consistent with those reported by Tsioutis et al. (*7*); high fever and intense headaches were the most common clinical features. Most of the patients fulfilled the criterion for FID. There were differences by age groups. The presence of a rash was rare among elderly patients, as reported (*12*,*13*). This finding makes the diagnostic utility of the classic triad of fever, headache, and rash somewhat debatable, especially for older patients. Furthermore, patients <20 years of age sometimes showed a clinical profile indistinguishable from that for infectious mononucleosis associated with pharyngitis, visceromegaly, lymphadenopathy, and atypical lymphocytosis.

The most common finding for blood counts was thrombocytopenia (127/218, 58.3%). A prolonged prothrombin time was common. No association was observed between a prolonged prothrombin time and complications, which is in contrast to the results of Chang et al. (*14*). Hypertransaminasemia was the most common serum alteration, which reached values typical for viral, toxic, or ischemic hepatitis ​​in 10% of case-patients. However, clinical hepatitis, with the presence of hepatomegaly and increased levels of bilirubin, was much less frequent.

The higher incidence of renal damage for patients with murine typhus in the Canary Islands has been reported (*15*). This differential finding could be caused by specific strains of *R. typhi* that have a particular tropism, although there is no solid evidence to confirm this possibility.

Transient IgM titers against other microorganisms in admission serum samples are common. Obtaining 2 independent samples during an interval of 2 weeks is essential to avoid false-negative results or misdiagnoses.

A limitation of this study is its retrospective design, although based on an established protocol. Another limitation is the possibility of cross-reactivity; cross-reactivity is common in rickettsial diseases, and some cases diagnosed as murine typhus may have been caused by other rickettsial species. A third limitation is use of a single serum sample as a diagnostic criterion, which although used in most clinical case series is not rigorous, and previous exposure to pathogens as the cause of seroreactivity cannot be completely ruled out. However, the relatively high IgM cutoff point and the absence of relevant differences between patients given a diagnosis by using 1 sample and those with confirmed seroconversion support the data presented.

Murine typhus is a major cause of FID in the Canary Islands. Complications are frequent, especially in the elderly, usually with renal, hepatic, respiratory, or central nervous system involvement. These results should help raise awareness among physicians about the need to identify cases earlier, start treatment promptly, and thus improve clinical outcomes.

Appendix Additional information on murine typhus in Canary Islands, Spain, 1999–2015.
