# Supplementary material for: Murine Typhus in Canary Islands, Spain, 1999–2015
Source: Emerg Infect Dis. 2021 Feb;27(2):570–3. doi: 10.3201/eid2702.191695 (PMC7853564; doi:10.3201/eid2702.191695)
Supplement: Appendix — Additional information on murine typhus in Canary Islands, Spain, 1999–2015. [file 19-1695-Techapp-s1.pdf]

Article DOI: https://doi.org/10.3201.eid2702.191695

Murine Typhus in Canary Islands, Spain, 1999–2015

Appendix

Table 1. Cutoff values for patients who have murine typhus in Canary Islands, Spain, 1999–2015*

Laboratory findings	Cutoff values
Anemia	<12 g/dL Hb in women, <13 g/dL Hb in men
Leukopenia	<4,000 /µL
Leukocytosis	>11,000/µL
Thrombocytopenia	<150,000/µL
Increased ESR	Upper limit of normality calculated according to age and sex
Decrease in Quick ratio	<80%
Increased aPTT ratio	>1.2
Increased plasma creatinine	>1.2 mg/dL
Hyponatremia	<135 mEq/L
Plasma creatine kinase	>232 U/L
Increased plasma urea	>40 mg/dL
Increased plasma LDH	>190 U/L
Increased plasma ALT	>35 U/L
Increased plasma AST	>35 U/L
Increased plasma alkaline phosphatase	>136 U/L
Increased plasma GGT	>85 U/L
Microhematuria	>5 RBCs/mm^3
Proteinuria	Positive urine test strip
Leukocyturia	>10 WBCs/mm^3

*ALT, alanine aminotransferase; aPTT: activated partial thromboplastin time; AST, aspartate aminotransferase; ESR, erythrocyte sedimentation rate; GGT, γ-glutamyl transpeptidase; LDH, lactate dehydrogenase; PT, prothrombin time; WBCs, white blood cells.

Table 2. Complication criteria for patients who have murine typhus in Canary Islands, Spain, 1999–2015*

Complication	Criteria
Hepatitis	ALT or AST increase ≥10 times the upper limit of reference range
Acute renal failure	Increased creatinine x 1.5 or ≥0.3 mg/dL from baseline
Meningitis	Suggestive signs or symptoms AND pleocytosis (>10 WBCs/mm^3) in cerebrospinal fluid
Pneumonia	Suggestive signs or symptoms AND development of pulmonary infiltrates on radiograph

*ALT, alanine aminotransferase; AST, aspartate aminotransferase; WBCs, white blood cells.

Table 3. Complications for patients who have murine typhus in Canary Islands, Spain, 1999–2015

Complication	Total, no. (%)	IgM ≥1:1,280, no. (%)*	4x IgG titer increase, no. (%)†	p-value
Hepatitis	22 (10)	5 (6.9)	17 (14.4)	0.35
Acute renal failure	21 (9.8)	11 (15.9)	10 (6.8)	0.05
Meningitis	12 (5.6)	4 (5.8)	8 (5.5)	0.99
Pneumonia	9 (4.2)	4 (5.8)	5 (3.4)	0.47

*Patients given a diagnosis by testing of 1 sample (criterion 1).
†Patients given a diagnosis by testing of 2 consecutive samples (criterion 2).

Table 4. Case series patients who have murine typhus in Canary Islands, Spain, 1999–2015

Characteristic	Bernabeu (*1*), Sevilla, 1999	Miguélez (*2*), Tenerife, 2003	Hernández (*3*), Gran Canaria, 2003	This study, Gran Canaria, 2020
No. patients	104	32	22	221
Design	Prospective	Retrospective	Retrospective	Retrospective
Distribution by season	Summer–fall	No predominance	Summer	Summer–fall
Mean age, y	37.9	49	28	40
Sex, M:F	1.2:1	1.91:1	21:1	2.75:1
Cases in urban areas, %	63.5	21.9	Unknown	91.4

Table 5. Main series of single-center cases of murine typhus worldwide*

Study	Country	Year	Design	No. patients	Mean age, y	M:F
Taylor (*4*)	Texas (USA)	1986	Retrospective	200	35	1:1
Dumler (*5*)	Texas (USA)	1991	Retrospective	80	48	1:1.5
Silpapojakul (*6*)	Thailand	1992	Retrospective	137	Unknown	1.3:1
Tselentis (*7*)	Greece	1992	Retrospective	49	Unknown	Unknown
Gikas (*8*)	Greece	2004	Prospective	87	39	1.4:1
Phongmany (*9*)	Laos	2006	Prospective	41	40	1.7:1
Punda-Polic (*10*)	Croatia	2007	Prospective	57	38	1:1
Zimmerman (*11*)	Nepal	2008	Prospective	50	28	1:1.8
Adjemian (*12*)	Texas (USA)	2010	Prospective	53	39	1.3:1
Paris (*13*)	Laos	2011	Prospective	55	31	Unknown
Chaliotis (*14*)	Greece	2012	Prospective	90	43	2.1:1
Chang (*15*)	Taiwan	2012	Retrospective	81	50	2.1:1
Psaroulaki (*16*)	Cyprus	2012	Prospective	193	37	1.9:1
Walter (*17*)	France	2012	Retrospective	32	42	1:1
Anyfantakis (*18*)	Greece	2013	Retrospective	165	Unknown	1.3:1
Znazen (*19*)	Tunisia	2013	Retrospective	43	41	Unknown
Tsioutis (*20*)	Greece	2014	Prospective	49	42	1.6:1
Aouam (*21*)	Tunisia	2015	Retrospective	73	33	1.8:1
Hamaguchi (*22*)	Vietnam	2015	Retrospective	193	47	2.6:1
Grouteau (*23*)	France	2019	Prospective	61	35	1:1

*Pediatric, Spanish, and studies including <30 cases were excluded.

References

1. Bernabeu-Wittel M, Pachón J, Alarcón A, López-Cortés LF, Viciana P, Jiménez-Mejías ME, et al. Murine typhus as a common cause of fever of intermediate duration: a 17-year study in the south of Spain. Arch Intern Med. 1999;159:872–6. PubMed https://doi.org/10.1001/archinte.159.8.872

2. Miguélez M, Laynez P, Linares M, Hayek M, Abella L, Marañez I. Murine typhus in Tenerife. Clinicoepidemiological study and differential clinical features with Q fever [in Spanish]. Med Clin (Barc). 2003;121:613–5. PubMed

3. Hernández Cabrera M, Angel-Moreno A, Santana E, Bolaños M, Francès A, Martín-Sánchez MS, et al. Murine typhus with renal involvement in Canary Islands, Spain. Emerg Infect Dis. 2004;10:740–3. PubMed https://doi.org/10.3201/eid1004.030532

4. Taylor JP, Betz TG, Rawlings JA. Epidemiology of murine typhus in Texas. 1980 through 1984. JAMA. 1986;255:2173–6. PubMed https://doi.org/10.1001/jama.1986.03370160071029

5. Dumler JS, Taylor JP, Walker DH. Clinical and laboratory features of murine typhus in south Texas, 1980 through 1987. JAMA. 1991;266:1365–70. PubMed https://doi.org/10.1001/jama.1991.03470100057033

6. Silpapojakul K, Mitarnun W, Ovartlarnporn B, Chamroonkul N, Khow-Ean U. Liver involvement in murine typhus. QJM. 1996;89:623–9. PubMed https://doi.org/10.1093/qjmed/89.8.623

7. Tselentis Y, Babalis TL, Chrysanthis D, Gikas A, Chaliotis G, Raoult D. Clinicoepidemiological study of murine typhus on the Greek island of Evia. Eur J Epidemiol. 1992;8:268–72. PubMed https://doi.org/10.1007/BF00144812

8. Gikas A, Doukakis S, Pediaditis J, Kastanakis S, Manios A, Tselentis Y. Comparison of the effectiveness of five different antibiotic regimens on infection with *Rickettsia typhi*: therapeutic data from 87 cases. Am J Trop Med Hyg. 2004;70:576–9. PubMed https://doi.org/10.4269/ajtmh.2004.70.576

9. Phongmany S, Rolain JM, Phetsouvanh R, Blacksell SD, Soukkhaseum V, Rasachack B, et al. Rickettsial infections and fever, Vientiane, Laos. Emerg Infect Dis. 2006;12:256–62. PubMed https://doi.org/10.3201/eid1202.050900

10. Punda-Polić V, Luksić B, Capkun V. Epidemiological features of Mediterranean spotted fever, murine typhus, and Q fever in Split-Dalmatia County (Croatia), 1982–2002. Epidemiol Infect. 2008;136:972–9. PubMed https://doi.org/10.1017/S0950268807009491

11. Zimmerman MD, Murdoch DR, Rozmajzl PJ, Basnyat B, Woods CW, Richards AL, et al. Murine typhus and febrile illness, Nepal. Emerg Infect Dis. 2008;14:1656–9. PubMed https://doi.org/10.3201/eid1410.080236

12. Adjemian J, Parks S, McElroy K, Campbell J, Eremeeva ME, Nicholson WL, et al. Murine typhus in Austin, Texas, USA, 2008. Emerg Infect Dis. 2010;16:412–7. PubMed https://doi.org/10.3201/eid1603.091028

13. Paris DH, Chansamouth V, Nawtaisong P, Löwenberg EC, Phetsouvanh R, Blacksell SD, et al. Coagulation and inflammation in scrub typhus and murine typhus: a prospective comparative study from Laos. Clin Microbiol Infect. 2012;18:1221–8. PubMed https://doi.org/10.1111/j.1469-0691.2011.03717.x

14. Chaliotis G, Kritsotakis EI, Psaroulaki A, Tselentis Y, Gikas A. Murine typhus in central Greece: epidemiological, clinical, laboratory, and therapeutic-response features of 90 cases. Int J Infect Dis. 2012;16:e591–6. PubMed https://doi.org/10.1016/j.ijid.2012.03.010

15. Chang K, Chen YH, Lee NY, Lee HC, Lin CY, Tsai JJ, et al. Murine typhus in southern Taiwan during 1992–2009. Am J Trop Med Hyg. 2012;87:141–7. PubMed https://doi.org/10.4269/ajtmh.2012.11-0465

16. Psaroulaki A, Christou C, Chochlakis D, Tsiligianni I, Sandalakis V, Georgalis L, et al. Murine typhus in Cyprus: a 9-year survey. Trans R Soc Trop Med Hyg. 2012;106:489–95. PubMed https://doi.org/10.1016/j.trstmh.2012.02.010

17. Walter G, Botelho-Nevers E, Socolovschi C, Raoult D, Parola P. Murine typhus in returned travelers: a report of thirty-two cases. Am J Trop Med Hyg. 2012;86:1049–53. PubMed https://doi.org/10.4269/ajtmh.2012.11-0794

18. Anyfantakis D, Doukakis S, Papadakis M, Triantafyllidou D, Bambili K, Polimili G, et al. Liver function test abnormalities in murine typhus in Greece: a retrospective study of 165 cases. Infez Med. 2013;21:207–10. PubMed

19. Znazen A, Hammami B, Mustapha AB, Chaari S, Lahiani D, Maaloul I, et al. Murine typhus in Tunisia: a neglected cause of fever as a single symptom. Med Mal Infect. 2013;43:226–9. PubMed https://doi.org/10.1016/j.medmal.2013.02.007

20. Tsioutis C, Chaliotis G, Kokkini S, Doukakis S, Tselentis Y, Psaroulaki A, et al. Murine typhus in elderly patients: a prospective study of 49 patients. Scand J Infect Dis. 2014;46:779–82. PubMed https://doi.org/10.3109/00365548.2014.943283

21. Aouam A, Toumi A, Ben Brahim H, Loussaief C, Jelliti B, Ben Romdhane F, et al. Epidemiological, clinical and laboratory features of murine typhus in central Tunisia. Med Mal Infect. 2015;45:124–7. PubMed https://doi.org/10.1016/j.medmal.2015.01.009

22. Hamaguchi S, Cuong NC, Tra DT, Doan YH, Shimizu K, Tuan NQ, et al. Clinical and epidemiological characteristics of scrub typhus and murine typhus among hospitalized patients with acute undifferentiated fever in northern Vietnam. Am J Trop Med Hyg. 2015;92:972–8. PubMed https://doi.org/10.4269/ajtmh.14-0806

23. Grouteau G, Lancelot O, Bertolotti A, Poubeau P, Manaquin R, Foucher A, et al. Emergence of murine typhus in La Réunion, France, 2012–2017. Med Mal Infect. 2020;50:22–7. PubMed https://doi.org/10.1016/j.medmal.2019.06.003
